# Oxr1 improves pathogenic cellular features of ALS-associated FUS and TDP-43 mutations

**DOI:** 10.1093/hmg/ddv104

**Published:** 2015-03-19

**Authors:** Mattéa J. Finelli, Kevin X. Liu, Yixing Wu, Peter L. Oliver, Kay E. Davies

**Affiliations:** MRC Functional Genomics Unit, Department of Physiology, Anatomy and Genetics, University of Oxford, Parks Road, Oxford OX1 3PT, UK

## Abstract

Amyotrophic lateral sclerosis (ALS) is a neurodegenerative disease characterized by the loss of motor neuron-like cells. Mutations in the RNA- and DNA-binding proteins, fused in sarcoma (FUS) and transactive response DNA-binding protein 43 kDa (TDP-43), are responsible for 5–10% of familial and 1% of sporadic ALS cases. Importantly, aggregation of misfolded FUS or TDP-43 is also characteristic of several neurodegenerative disorders in addition to ALS, including frontotemporal lobar degeneration. Moreover, splicing deregulation of FUS and TDP-43 target genes as well as mitochondrial abnormalities are associated with disease-causing FUS and TDP-43 mutants. While progress has been made to understand the functions of these proteins, the exact mechanisms by which FUS and TDP-43 cause ALS remain unknown. Recently, we discovered that, in addition to being up-regulated in spinal cords of ALS patients, the novel protein oxidative resistance 1 (Oxr1) protects neurons from oxidative stress-induced apoptosis. To further understand the function of Oxr1, we present here the first interaction study of the protein. We show that Oxr1 binds to Fus and Tdp-43 and that certain ALS-associated mutations in Fus and Tdp-43 affect their Oxr1-binding properties. We further demonstrate that increasing Oxr1 levels in cells expressing specific Fus and Tdp-43 mutants improves the three main cellular features associated with ALS: cytoplasmic mis-localization and aggregation, splicing changes of a mitochondrial gene and mitochondrial defects. Taken together, these findings suggest that OXR1 may have therapeutic benefits for the treatment of ALS and related neurodegenerative disorders with TDP-43 pathology.

## Introduction

Amyotrophic lateral sclerosis (ALS) is a neurodegenerative disease characterized by the loss of motor neuron-like cells, which leads to progressive muscle atrophy and death within 1–5 years. Genetic mutations have been found to cause both sporadic (sALS) and familial ALS (fALS) cases; in addition to mutations in superoxide dismutase 1 (SOD1) and C9ORF72, fused in sarcoma (FUS) and transactive response DNA-binding protein 43 kDa (TDP-43) are responsible for ∼5–10% of fALS cases and ∼1% of sALS cases (1–11). FUS and TDP-43 are DNA- and RNA-binding proteins that modulate transcriptional regulation, pre-RNA splicing and micro-RNA processing (12,13). While FUS and TDP-43 are normally located in the nucleus, FUS-positive and TDP-43-positive cytoplasmic inclusions are pathological hallmarks of most non-SOD1 sALS cases and a related neurological disorder, frontotemporal lobar degeneration (FTLD) (9,14–17). Therefore, the identification of factors that ameliorate the mis-localization of FUS and TDP-43 mutants could be one of the avenues worth pursuing for the design of novel therapeutic strategies for ALS.

While the exact consequence of ALS mutations on FUS and TDP-43 cytoplasmic localization remains unknown, increasing evidence suggests that ALS mutant FUS and TDP-43 are associated with increased neuronal cell death *in vitro* and disease severity in ALS patients (18–27). Recent work has sought to understand mechanisms governing nucleo-cytoplasmic transport and aggregation of FUS and TDP-43 mutants. While pathways that underlie TDP-43 localization are still not well understood, arginine methylation by protein arginine *N*-methyltransferase 1 (PRMT1) and direct interaction with transportin regulate FUS shuttling between the nucleus and cytoplasm (28–33). Furthermore, stress, particularly oxidative stress, recruits wild-type and ALS mutant FUS and TDP-43 to cytoplasmic stress granules (34–42). Oxidative stress is a central feature of ALS (43) and stress granule markers have been found to co-localize with FUS-positive and TDP-43-positive inclusions in the spinal cord of ALS patients (32,34,44), suggesting long-term oxidative stress is associated with pathological inclusions of FUS and TDP-43 in both fALS and sALS.

Mitochondrial dysfunction has also been described in cells from ALS patients (45) as well as in animal and cellular models of ALS-FUS and TDP-43 mutants. Both wild-type and mutant TDP-43 co-localize with mitochondria in yeast and mammalian cells and defects in mitochondria structure and dynamics are observed in cells over-expressing ALS-TDP-43 mutants (28,46–50). In addition, mitochondrial function is affected by ALS mutations in TDP-43: an inverse correlation between respiratory activity and toxicity of the mutants is observed in yeast (50), while the mitochondrial membrane potential is reduced in motor neurons transfected with a TDP-43 mutant (46). Microinjection of ALS-FUS mutants in mouse motor neurons also causes a significant shortening of mitochondria, a feature that is similarly observed in other ALS models (21,28).

We previously reported that oxidation resistance 1 (Oxr1) is up-regulated in the spinal cord of end-stage ALS patients as well as in a pre-symptomatic mouse model of ALS (51). Mice lacking *Oxr1* display neurodegeneration and we demonstrated that the levels of Oxr1 were critical for neuronal survival under oxidative conditions (51); our findings suggested that Oxr1 could serve as a neuroprotective factor in neurodegenerative diseases (51). Here, we investigated the role of Oxr1 in non-pathological conditions as well as in the context of ALS. Using an unbiased proteomic approach, we uncovered Oxr1 binding partners under basal and oxidative stress conditions and identified novel functions for Oxr1. In particular, Oxr1 binds to Fus and Tdp-43, and over-expression of Oxr1 reduces cytoplasmic mis-localization of ALS-Fus and Tdp-43 mutants. In addition, we show that over-expression of Oxr1 restores splicing of *Mtfr1*, a gene involved in mitochondrial fission, and improves mitochondrial morphology in motor neuron-like cells expressing Tdp-43 M337V mutant. Together, these findings suggest that OXR1 plays an important role in regulating FUS and TDP-43 pathology in neurodegenerative disease.

## Results

### Transcription of Oxr1 isoforms is regulated under oxidative stress

**Figure 1.**
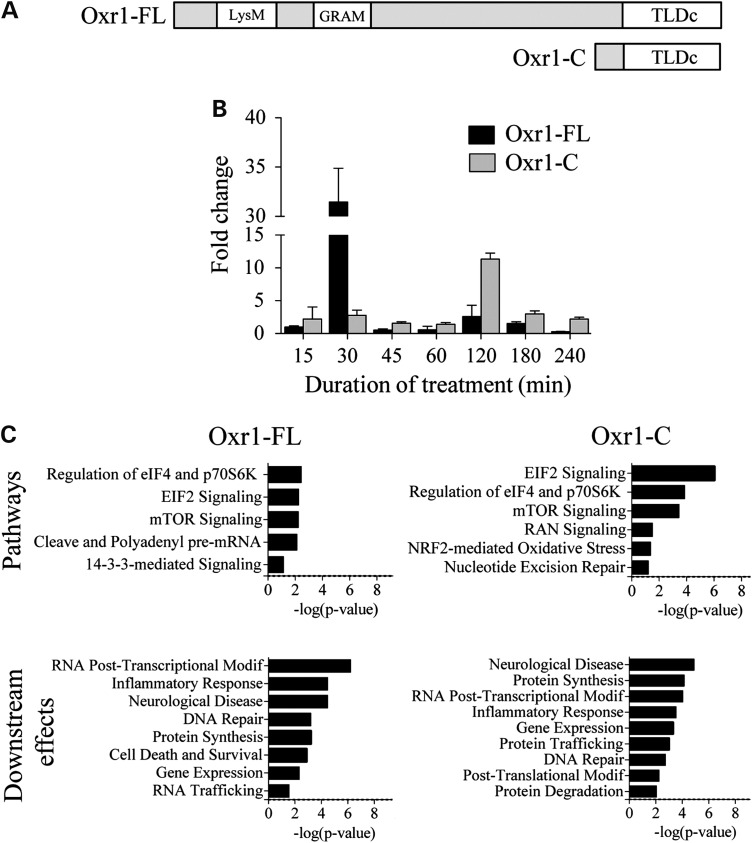
Oxr1 isoforms are multifunctional proteins. (**A**) Schematic diagram (not to scale) of full-length Oxr1 (Oxr1-FL) and the short Oxr1 isoform (Oxr1-C). Oxr1-C consists predominantly of the TLDc domain, whereas Oxr1-FL also contains a LysM (Lysin motif) and a GRAM domain. (**B**) Oxr1-FL and Oxr1-C levels by real-time PCR in N2a cells treated with 150 m H_2_O_2_ for various durations. Oxr1 expression is induced under OS at different stage of the OS-response (*n* = 3). (**C**) Common functional pathways of the proteins co-immunoprecipitated with Oxr1-FL and Oxr1-C using Ingenuity Pathway Analysis.

Previous studies have demonstrated that Oxr1 is induced by oxidative stress and over-expression of the gene can be protective under cellular stress *in vitro* and *in vivo* (51,52). The gene is expressed as several isoforms, all containing a C-terminal TLDc domain, which is highly conserved among species and present in all eukaryotes (Fig. 1A) (53,54). The TLDc domain has also been shown to prevent oxidative damage in various organisms (51,52,55); however, its mechanism of action remains unclear. Importantly, the shortest of these isoforms (Oxr1-C), almost entirely made-up of the TLDc domain, is highly expressed in the nervous system and is sufficient to protect neurons against oxidative stress (51). In order to investigate whether these different Oxr1 isoforms have independent functions, we first examined when the Oxr1 full-length (Oxr1-FL) and shortest (Oxr1-C) isoforms are induced during the oxidative stress response. After treating non-transfected neuronal Neuro-2a (N2a) cells with H_2_O_2_ for various durations to induce cellular oxidative stress, we examined Oxr1-FL and Oxr1-C mRNA levels by qRT–PCR. Over the 240 min time-course of the experiment, both transcripts showed a significant up-regulation; at 30 min for Oxr1-FL (∼30-fold) and at 120 min for Oxr1-C (∼11-fold) (Fig. 1B). These data show that Oxr1 isoforms are not necessarily co-regulated, suggesting that they may have specific functions or act at different stages of the oxidative stress response.

### Oxr1 isoforms differentially interact with ALS-associated proteins involved in RNA processing

In an attempt to elucidate the molecular functions of Oxr1 in neurons, we co-immunoprecipitated binding partners in N2a cells, a neuronal cell line, expressing either tagged Oxr1-FL or Oxr1-C or an empty control vector in the presence or absence of oxidative stress treatment. The associated binding proteins were then identified by mass spectrometry. Oxr1-C had fewer binding partners in non-treated (3 in total) compared with H_2_O_2_-treated conditions (38) (Table 1). In contrast, Oxr1-FL had a similar number of binding partners in non-treated (16) and treated (13) conditions (Table 1).

**Table 1.** Interacting proteins after 4-h H_2_O_2_ treatment

Symbol	Entrez gene name	Type(s)
Oxr1-FL		
BTBD6	BTB (POZ) domain containing 6	Other
DDX3X	DEAD (Asp-Glu-Ala-Asp) box helicase 3, X-linked	Enzyme
Ewsr1	Ewing sarcoma breakpoint region 1	Other
FARSA	Phenylalanyl-tRNA synthetase, alpha subunit	Enzyme
FUS	Fused in sarcoma	Transcription regulator
Hnrnpa1	Heterogeneous nuclear ribonucleoprotein A1	Other
NONO	Non-POU domain containing, octamer-binding	Other
PABPN1	Poly(A) binding protein, nuclear 1	Enzyme
PAX6	Paired box 6	Transcription regulator
PTGR1	Prostaglandin reductase 1	Enzyme
RPS16	Ribosomal protein S16	Other
RPS19	Ribosomal protein S19	Other
VIM	Vimentin	Other
Oxr1-C		
APRT	Adenine phosphoribosyltransferase	Enzyme
ATP5B	ATP synthase, H+ transporting, mitochondrial F1 complex, β polypeptide	Transporter
BTBD6	BTB (POZ) domain containing 6	Other
CCT2	Chaperonin containing TCP1, subunit 2 (β)	Kinase
CCT6A	Chaperonin containing TCP1, subunit 6A (ζ 1)	Other
CCT7	Chaperonin containing TCP1, subunit 7 (η)	Other
CDKN2A	Cyclin-dependent kinase inhibitor 2A	Transcription regulator
DDX3X	DEAD (Asp-Glu-Ala-Asp) box helicase 3, X-linked	Enzyme
DNAJC19	DnaJ (Hsp40) homolog, subfamily C, member 19	Other
EEF1A1	Eukaryotic translation elongation factor 1 alpha 1	Translation regulator
ENO2	Enolase 2 (gamma, neuronal)	Enzyme
FARSA	Phenylalanyl-tRNA synthetase, alpha subunit	Enzyme
FUS	Fused in sarcoma	Transcription regulator
Gm11517	Ubiquitin A-52 residue ribosomal protein fusion product 1 pseudogene	Other
HNRNPA2B1	Heterogeneous nuclear ribonucleoprotein A2B1	
HNRNPC	Heterogeneous nuclear ribonucleoprotein C (C1/C2)	Other
LDHA	Lactate dehydrogenase A	Enzyme
NAP1L1	Nucleosome assembly protein 1-like 1	Other
NONO	Non-POU domain containing, octamer-binding	Other
PAX6	Paired box 6	Transcription regulator
POLR2H	Polymerase (RNA) II (DNA directed) polypeptide H	Enzyme
PRMT1	Protein arginine methyltransferase 1	Enzyme
PRPH	Peripherin	Other
RAN	RAN, member RAS oncogene family	Enzyme
RPL9	Ribosomal protein L9	Other
RPS10	Ribosomal protein S10	Other
RPS11	Ribosomal protein S11	Other
RPS24	Ribosomal protein S24	Other
RPS7	Ribosomal protein S7	Other
RSL1D1	Ribosomal L1 domain containing 1	Other
SLC25A3	Solute carrier family 25 (mitochondrial carrier; phosphate carrier), member 3	Transporter
SLC25A5	Solute carrier family 25 (mitochondrial carrier; adenine nucleotide translocator), member 5	Transporter
SNRPD1	Small nuclear ribonucleoprotein D1 polypeptide 16 kDa	Other
TARDBP	TAR DNA-binding protein 43	Transcription regulator
UBA52	Ubiquitin A-52 residue ribosomal protein fusion product 1	Enzyme
UMPS	Uridine monophosphate synthetase	Enzyme
VIM	Vimentin	Other
ZCCHC6	Zinc finger, CCHC domain containing 6	Enzyme
		

To investigate biological pathways in which the identified proteins participate, we performed *in silico* pathway analysis on the Oxr1 binding partners. We identified EIF2 and mTOR, two pathways involved in the oxidative stress response (56–58), which is in accordance with the known function of Oxr1 as an important regulator of neuronal sensitivity to oxidative stress (51,52,55,59,60). The Nrf2-mediated oxidative stress response pathway was also identified for Oxr1-C, specifically. A subsequent analysis of possible Oxr1 downstream effects predicted that Oxr1 isoforms affect DNA repair, confirming previous functional data (Fig. 1C) (51). Surprisingly, Oxr1 was predicted to affect other cellular functions, including RNA post-transcriptional modification, protein synthesis, and inflammatory response (Fig. 1C).

### Specific ALS-mutations alter Fus and Tdp-43 binding to Oxr1

**Figure 2.**
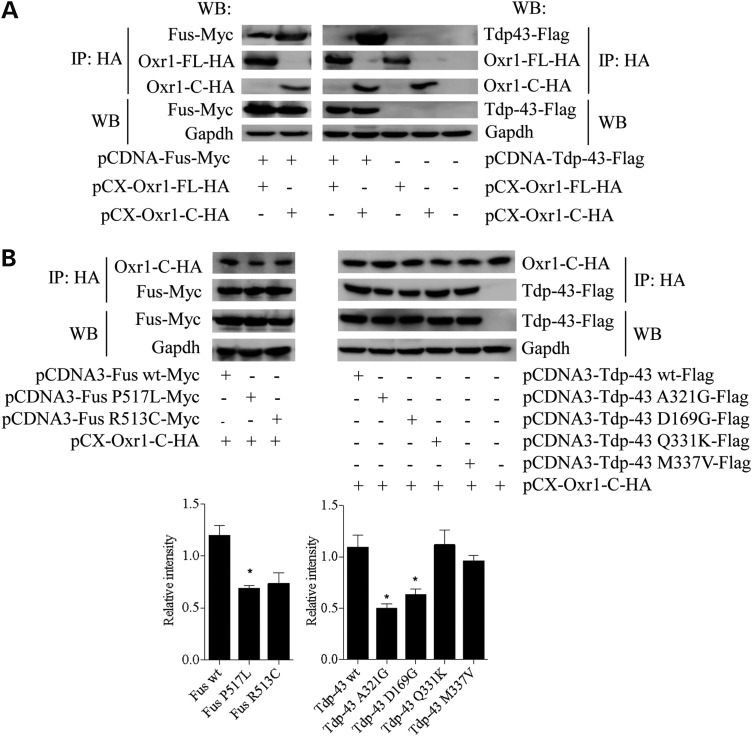
Oxr1 interacts with Fus and Tdp-43, two ALS-associated proteins. (**A**) Co-immunoprecipitation in N2a cells demonstrating that Fus binds to Oxr1-FL and -C while Tdp-43 binds to Oxr1-C only. (**B**) Co-immunoprecipitation of Oxr1-C with Fus and Tdp-43 wild-type and mutants (Fus P571L, Fus R513C, Tdp-43 A321G, Tdp-43 D169G, Tdp-43 Q331K and Tdp-43 M337V) in co-transfected N2a cells and western blot quantification. In A and B, the first three bands represent proteins immunoprecipitated with anti-HA beads, while the two last bands represent direct protein extracts to control for equal amount of tagged-proteins used per co-immunoprecipitation. Statistical significance was determined by one-way ANOVA (*n* = 3); *P < 0.05.

From this data set, we identified two DNA- and RNA-binding proteins, fused in sarcoma (Fus) and transactive response DNA-binding protein 43 kDa (Tdp-43) as Oxr1 binding partners. Given that FUS and TDP-43 mutations are associated with ALS and that OXR1 may be neuroprotective in this disease (9,11,14,15,51,61), we chose to investigate in more detail the interaction between Oxr1, Fus and Tdp-43. By co-immunoprecipitation in N2a cells co-expressing Myc-tagged Fus or Flag-tagged Tdp-43 with Ha-tagged Oxr1, we first confirmed that Fus binds to both Oxr1-FL and Oxr1-C, while Tdp-43 binds only to Oxr1-C (Fig. 2A). We next investigated whether pathogenic ALS-associated mutations in Fus and Tdp-43 influenced the binding to Oxr1. We co-expressed Oxr1-C with either wild-type or Fus (P517L and R513C) or Tdp-43 (A321G, D169G, Q331K and M337V) mutants (11,61–63). We first confirmed that these ALS-associated mutations did not affect the expression level of the constructs, both at the mRNA and protein level (Fig. 2B and Supplementary Material, Fig. S3A). Interestingly, co-immunoprecipitation showed that the interaction between Oxr1-C and Fus or Tdp-43 was altered by a specific subset of ALS-associated mutations (Fig. 2B). In particular, binding of Oxr1-C to Fus P517L, Tdp-43 A321G and Tdp-43 D169G is significantly decreased by 50.7, 59.9 and 47.0%, respectively, when compared with the interaction between Oxr1 and wild-type Fus or Tdp-43. Thus, these data suggest that ALS-Fus and -Tdp-43 mutations alter the binding of these proteins to Oxr1-C.

### Oxr1 reduces cytoplasmic aggregation of ALS-Fus and Tdp-43 mutants

**Figure 3.**
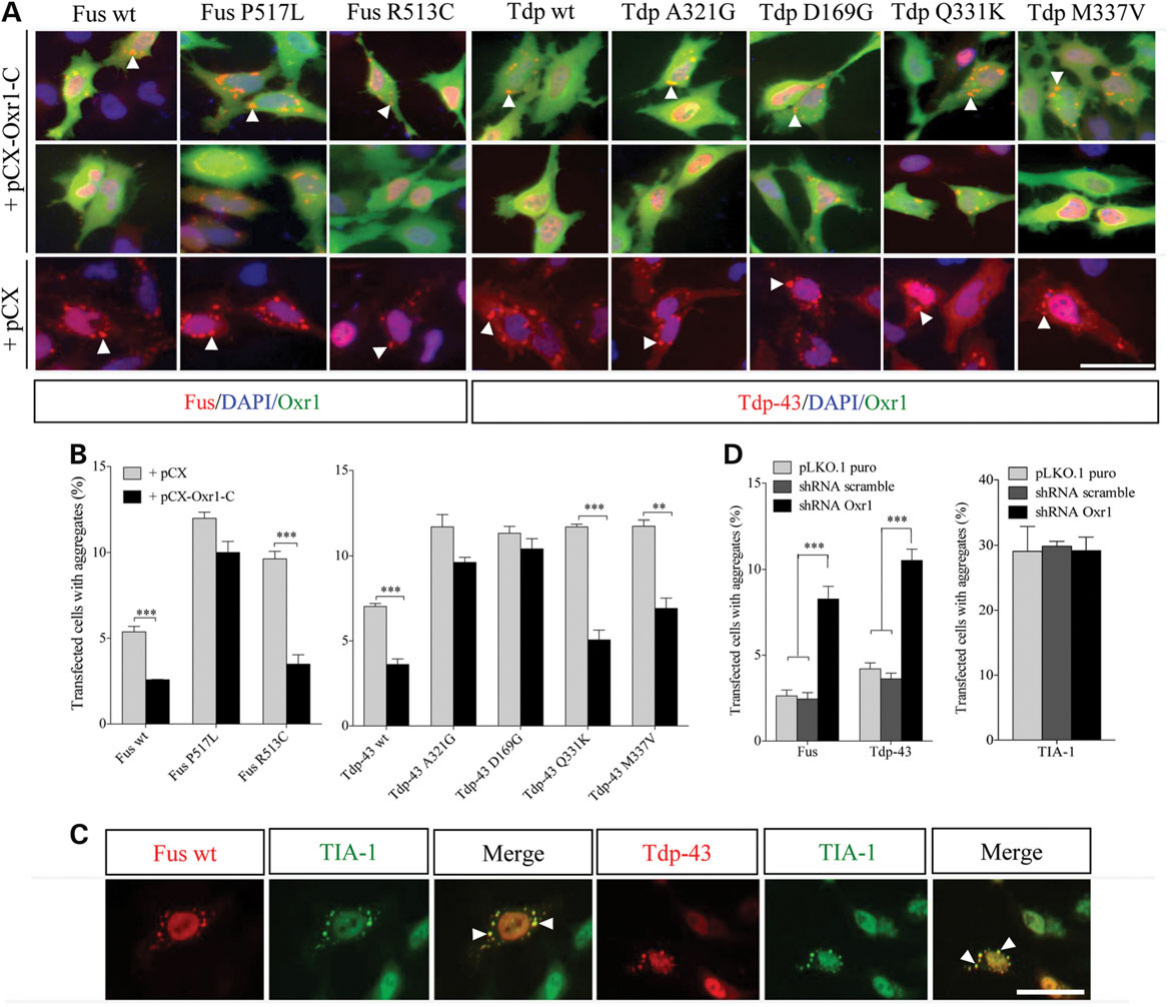
Over-expression of Oxr1-C decreases cytoplasmic aggregation of Fus and Tdp-43 wild-type and mutant under oxidative stress. (**A** and **B**) Representative images of cytoplasmic aggregation of Fus and Tdp-43 wild-type and mutants and quantification of cells forming aggregates under H_2_O_2_ treatment. Over-expression of Oxr1-C in HeLa cells significantly reduces aggregate formation for Fus wild-type (wt), Fus R513C, Tdp-43 wild-type (Tdp wt), Tdp-43 Q331K and Tdp-43 M337V. Scale bars: 25 μm. Statistical significance was determined by two-tailed unpaired Student's *t*-test (*n* = 3); **P < 0.01 and ***P < 0.001. (**C**) Representative images of Fus and Tdp-43 cytoplasmic aggregation under H_2_O_2_ treatment. Fus and Tdp-43 are recruited to TIA-1-positive stress granules under H_2_O_2_ treatment. Scale bars: 25 μm. (**D**) Quantification of cells forming Fus and Tdp-43 aggregates or TIA-1-positive aggregates under H_2_O_2_ treatment when transfected with shRNA *Oxr1* or control constructs. Scale bars: 25 μm. Statistical significance between co-transfection with pLKO.1 puro, shRNA scramble or shRNA *Oxr1* was determined by one-way ANOVA (*n* = 4); ***P < 0.001. Arrowheads indicate aggregates.

Recent studies have shown that ALS-mutations cause mis-localization of Fus and Tdp-43 to cytoplasmic inclusions, particularly under oxidative stress, a major component of ALS pathology (32,34–42,44,62). We showed previously that Oxr1-C protects neurons against oxidative stress-induced damage and apoptosis (51), thus we investigated whether Oxr1-C affects cellular localization of wild-type and Fus and Tdp-43 mutants under similar oxidative stress conditions. First, we confirmed that both wild-type and Fus and Tdp-43 mutants form cytoplasmic inclusions under exogenous peroxide treatment (Fig. 3A). Indeed, consistent with previous reports, we found that under H_2_O_2_-induced stress, wild-type and Fus and Tdp-43 mutants form cytoplasmic aggregates that preferentially localize to stress granules, as labelled by the marker cytotoxic granule-associated RNA-binding protein (TIA-1) (Fig. 3B and C and Supplementary Material, Fig. S1A) (32,34–42).

We next examined whether modulating Oxr1 levels could alter the cellular localization of wild-type or ALS-linked Fus and Tdp-43 mutants under oxidative stress. Interestingly, shRNA knockdown of all endogenous *OXR1* isoforms by ∼87.9% results in increased percentage of cells with Fus- and Tdp-43-positive cytoplasmic inclusions (Fig. 3D). We confirmed that the percentage of cells with TIA-1-positive stress granules is not affected by knock-down of *OXR1* or over-expression of Oxr1 (Fig. 3D), suggesting that Oxr1-C itself does not affect stress granule formation. Conversely, over-expression of Oxr1-C with wild-type Fus and Tdp-43 significantly reduces the accumulation of Fus- and Tdp-43-positive cytoplasmic inclusions after H_2_O_2_ treatment (Fig. 3A and B). In addition, we found that over-expression of Oxr1-C significantly decreases cytoplasmic aggregation of Fus R513C, Tdp-43 Q331K and Tdp-43 M337V mutants (Fig. 3B). Conversely, recruitment of Fus P517L, Tdp-43 A321G and Tdp-43 D169G to cytoplasmic inclusions is not altered by Oxr1-C over-expression; importantly, these three mutants also showed a markedly reduced interaction with Oxr1-C (Figs. 2B and 3B). These findings were similarly repeated under treatment with arsenite, another chemical compound that induces oxidative stress (Supplementary Material, Fig. S1B). Taken together, these findings suggest that interaction between Oxr1 and Fus or Tdp-43 is important for proper cellular localization of Fus and Tdp-43 under oxidative stress, and over-expression of Oxr1 reduces mis-localization of certain ALS-Fus and Tdp-43 mutants.

### Oxr1-mediated reduction in inclusions positive for Fus and Tdp-43 mutants is dependent on arginine methylation, but not proteasome degradation or autophagy

**Figure 4.**
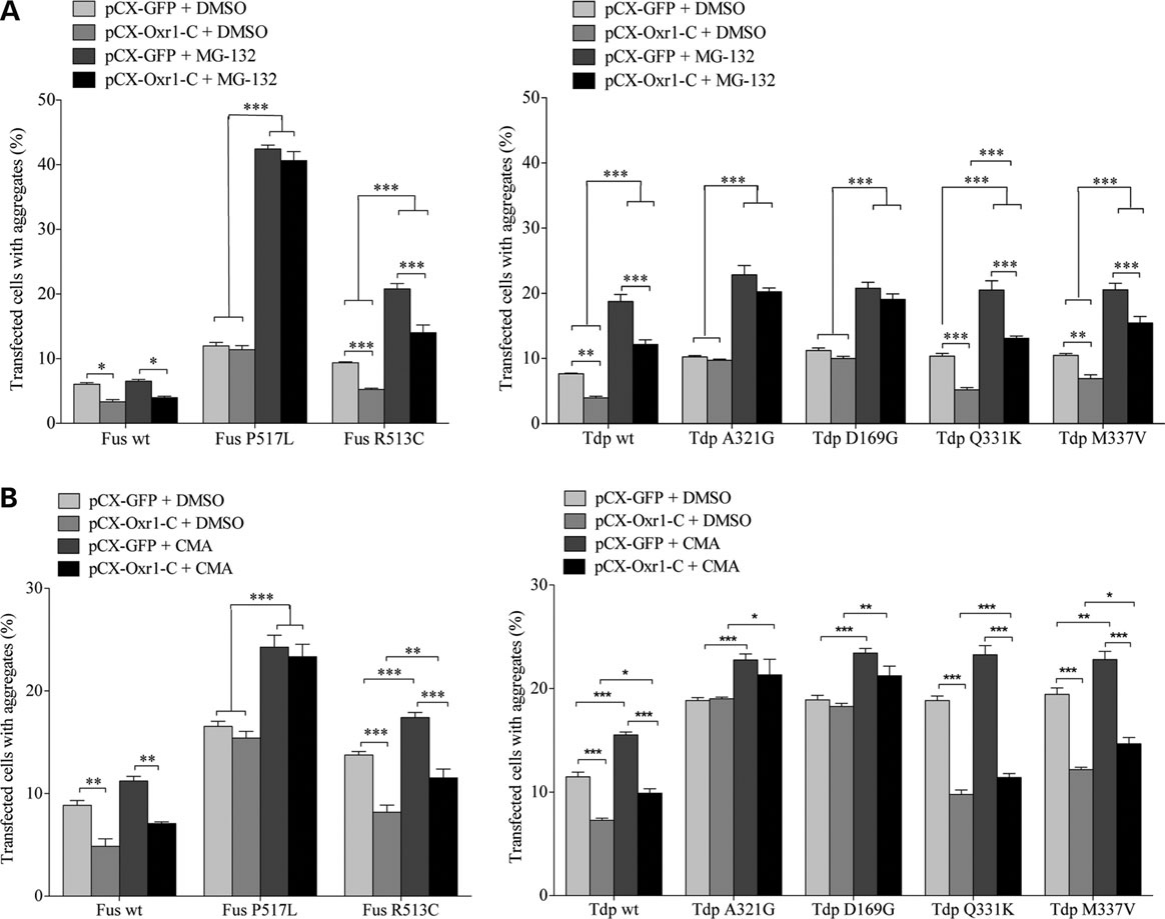
Reduction in cytoplasmic aggregation of wild-type and ALS-linked mutant Fus and Tdp-43 by Oxr1-C does not depend on proteasome degradation or autophagy. (**A** and **B**) Quantification of HeLa cells forming aggregates under H_2_O_2_ treatment when co-transfected with Fus or Tdp-43 wt and mutants, and with either pCX-GFP or Oxr1-C, after MG-132 treatment (**A**) or concanamycin A (CMA). (**B**) Statistical significance between co-transfection with GFP or Oxr1-C was determined by two-way ANOVA followed by Bonferroni's test (*n* = 3); *P < 0.05, **P < 0.01, and ***P < 0.001.

We next investigated mechanisms by which Oxr1 regulates Fus and Tdp-43 recruitment to stress granules. Both the proteasome and autophagy pathways are implicated in the clearance of Fus and Tdp-43 cytoplasmic inclusions (64–68). Moreover, our analysis suggested Oxr1-C is involved in protein degradation and functions upstream of the mTOR signalling, which is important for the proteasome and autophagy pathways (Fig. 1C) (69); therefore, we examined whether Oxr1-C-mediated regulation of Fus and Tdp-43 localization is dependent on these degradation pathways. To first test a role for the proteasome, we treated transfected cells with MG-132, a proteasome inhibitor, prior to oxidative stress treatment. Similar to previous findings, we found that MG-132 treatment significantly increases cytoplasmic inclusions of Tdp-43 mutants and, for the first time, Fus mutants, confirming that these aggregates are degraded, at least in part, by the proteasome (Fig. 4A) (68). Interestingly, under proteasome inhibition, over-expression of Oxr1-C is still able to significantly reduce the number of cytoplasmic inclusions positive for wild-type and Fus (R513C) and Tdp-43 (Q331K and M337V) mutants, demonstrating that the proteasome does not influence this particular function of Oxr1-C (Fig. 4A). We next examined the role of autophagy in Oxr1-mediated Fus and Tdp-43 localization by treating with concanamycin A, a commonly used inhibitor of autophagy, prior to oxidative stress treatment. Similarly, these data showed that although cytoplasmic Fus and Tdp-43 mutants are degraded through a mechanism involving autophagy, the ability of Oxr1-C to reduce these inclusions is not affected by concanamycin A treatment (Fig. 4B). Noticeably, in both the MG-132 and concanamycin A experiments, co-expression of Oxr1-C with Fus and Tdp-43 mutants that bind poorly to Oxr1-C (Fus P517L, Tdp-43 A321G and Tdp-43 D169G; Fig. 2B) does not show a reduction in Fus or Tdp-43 cytoplasmic inclusions, suggesting that the binding of Oxr1 to Fus and Tdp-43 is necessary to influence inclusion formation. Taken together, these data demonstrate that over-expression of Oxr1-C decreases Fus and Tdp-43 cytoplasmic inclusions in a manner that is independent of proteasome and autophagy protein degradation pathways but requires direct binding of Oxr1-C.

**Figure 5.**
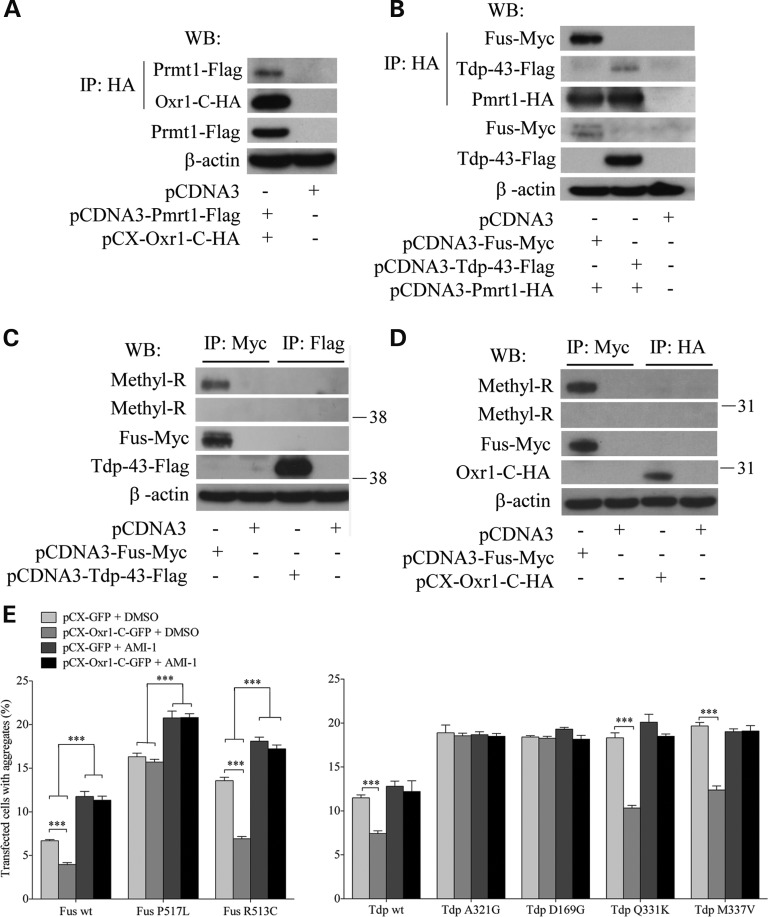
Reduction in cytoplasmic aggregation of wild-type and ALS-linked mutant Fus and Tdp-43 by Oxr1-C depends on arginine methylation. (**A**) Co-IP of Oxr1-C and Prmt1 in co-transfected N2a cells shows that Oxr1-C interacts with Prmt1. (**B**) Co-IP of Fus and Tdp-43 with Prmt1 in co-transfected N2a cells demonstrates Fus and Tdp-43 interact with Prmt1. (**C** and **D**) Arginine residues of Fus, but not of Tdp-43 (**C**) or Oxr1-C (**D**) are methylated. (**E**) Quantification of HeLa cells forming aggregates under H_2_O_2_ treatment when co-transfected with Fus wild-type (wt) and mutants (P517L and R513C) or Tdp-43 wild-type and mutants (A321G, D169G, Q331K and M337V), and with either GFP or Oxr1-C with and without AMI-1 treatment. Statistical significance between co-transfection with GFP or Oxr1-C was determined by two-way ANOVA followed by Bonferroni's test (*n* = 3); ***P < 0.001.

Previous studies have found that arginine methylation of Fus by Prmt1 regulates Fus nuclear localization (28–33). Because we identified Prmt1 as a binding partner of Oxr1-C, we examined whether Prmt1 plays a role in the mechanism underlying Oxr1-mediated cellular localization of Fus and Tdp-43 mutants. First, we confirmed that Oxr1-C, and not Oxr1-FL, interacts with Prmt1 by co-immunoprecipitation in N2a cells co-transfected with Prmt1 and Oxr1-C (Fig. 5A and Supplementary Material, Fig. S2A). Similarly, we confirmed that Fus binds to Prmt1 (Fig. 5B) (28,29,70,71); surprisingly, Tdp-43 also interacts with Prmt1 (Fig. 5B). Prmt1 is responsible for the majority of the arginine methylation events in the cell (72), so we next tested if Prmt1 methylates arginine residues on Fus, Tdp-43 and Oxr1-C. However, only Fus is methylated by Prmt1, which is consistent with a previous study (Fig. 5C and D) (28,29,31). To investigate whether Oxr1-C-mediated Fus and Tdp-43 cellular localization was dependent on Prmt1 activity, we treated transfected cells with AMI-1, a Prmt1-specific inhibitor (73), before H_2_O_2_ treatment. We confirmed that after blocking Prmt1 activity, methylation of endogenous Fus is decreased (Supplementary Material, Fig. S2B). However, methylation of over-expressed Fus is not affected by AMI-1 treatment (Supplementary Material, Fig. S2B). Furthermore, binding of over-expressed Oxr1-C with over-expressed Fus and Tdp-43 is not dependent on Prmt1 activity (Supplementary Material, Fig. S2C and D). However, we observed significantly increased number of cells with Fus-positive cytoplasmic aggregates after AMI-1 treatment, confirming that the presence of Prmt1 and Fus methylation is required for Fus localization to the nucleus (Fig. 5E) (28,29,31). Interestingly, inhibition of Prmt1 function also prevents Oxr1-C from significantly decreasing cytoplasmic aggregation of wild-type and ALS mutant Fus and Tdp-43 whose binding with Oxr1-C is not affected (Fig. 5E). Taken together, our findings suggest that the function of Oxr1-C in mediating cellular localization of Fus and Tdp-43 is dependent on Prmt1 function.

### Oxr1 restores splicing of *Mtfr1* affected by an ALS-linked Tdp-43 mutation

**Figure 6.**
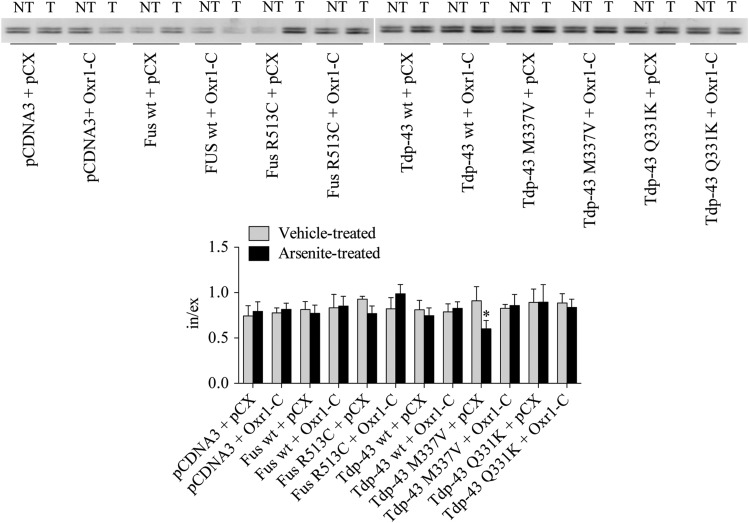
*Mtfr1* splicing defect in motor neurons over-expressing Tdp-43 M337 is rescued by Oxr1-C. NSC-34 cells were transfected with either Fus or Tdp-43 wild-type (wt) and mutants together with pCX empty vector or Oxr1-C for 24 h. Cells were either vehicle-treated (NT) or treated with arsenite (T) for 4 h. Representative RT–PCR of exon 4 of *Mtfr1* gene (top panel). Densitometry quantification of agarose gel signal intensity and calculation of the inclusion to exclusion ratio (in/ex) of exon 4 (bottom panel). Statistical significance between non-treated and treated conditions was determined by two-way ANOVA followed by Bonferroni's test (*n* = 3–6); *P < 0.05.

One known function of Fus and Tdp-43 is pre-mRNA splicing regulation (12,13). ALS-associated mutations in Tdp-43 cause splicing dysregulation in various cellular and animal models (74–83). As our analysis of Oxr1 binding partners predicted that Oxr1 has a function in RNA splicing (Fig. 1C), we investigated whether Oxr1 modulates Fus and Tdp-43-mediated splicing under basal and oxidative stress conditions in the motor neuron-like cell line, NSC-34. As Oxr1 is involved in the oxidative stress response (51,52,59,60), we hypothesized that Oxr1 may have a role in mitochondrial function. Thus, we studied splicing of *Slc1a2, Opa1, Mtfr1,* a set of mitochondrial genes, and *Mapt*, *Tia1, Taf1b* and *Eif4 h,* important genes for the oxidative stress response (84–87). Moreover, the selected genes are expressed in the grey matter of the mouse spinal cord (Allen Brain Atlas) and have been associated with ALS pathophysiology (36,39,74,88–91). No significant difference in splicing of the selected genes was detected in non-treated conditions for Fus and Tdp-43 wild-type and mutants (Supplementary Material, Fig. S3B). However, when cells were treated with arsenite to induce oxidative stress, we observed a slight but significant decrease in the ratio of exon exclusion to inclusion of *Mtfr1* only in cells transfected with Tdp-43 M337V (Fig. 6). Interestingly, co-transfection of Tdp-43 M337V with Oxr1-C restored normal splicing of *Mtfr1* under oxidative stress (Fig. 6). Importantly, we found no changes in splicing in non-treated or treated cells over-expressing only Oxr1-C, suggesting that Oxr1-C alone does not affect the splicing of *Mtfr1* (Fig. 6). Thus, our finding suggests that the ALS-associated Tdp-43 M337V mutation affects Tdp-43 function as splicing regulator of *Mtfr1* under oxidative stress*,* and that over-expression of Oxr1-C rescues abnormal splicing dysregulation associated with Tdp-43 M337V.

### Oxr1 restores mitochondrial morphology defects and dysfunction associated with an ALS-mutation in Tdp-43

**Figure 7.**
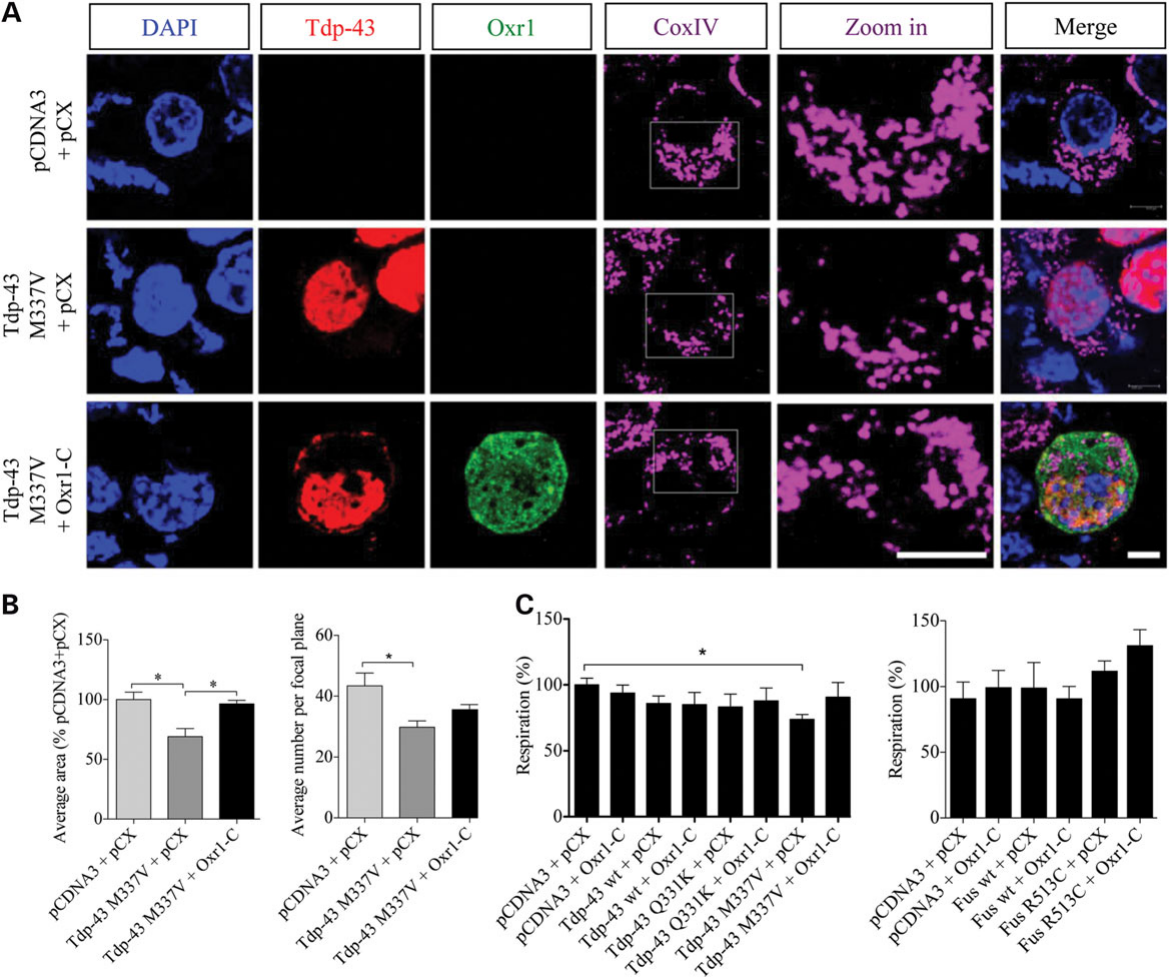
Oxr1 restores normal mitochondrial morphology and oxygen consumption in motor neurons expressing Tdp-43 M337V mutant. (**A**) Representative images of NSC-34 cells transfected with Flag-tagged Tdp-43 M337V with empty vectors (pCDNA3 + pCX) or HA-tagged Oxr1-C for 24 h. COXIV was used as a mitochondrial marker. Higher magnification (‘zoom in’) images of the fragmented mitochondrial structure in cells transfected with Tdp-43 M337V mutants are also shown. Scale bars: 5 μm in all panels. (**B**) The average mitochondrial area and average number of mitochondria were quantified; mitochondria in cells transfected with Tdp-43 M337V were smaller and fragmented when compared with cells transfected with empty vectors or co-transfected with Tdp-43 M337V and Oxr1-C (*n* = 3–4). (**C**) Oxygen consumption was quantified on NSC-34 cells transfected with the indicated vectors for 24 h. Statistical significance between cells transfected with empty vectors or Tdp-43 and Oxr1-C was determined by one-way ANOVA (*n* = 3–4); *P < 0.05.

*Mtfr1* is an important regulator of mitochondrial fission and Tdp-43 M337V has been shown to affect mitochondrial dynamics (46,92), thus we next investigated whether Oxr1 also ameliorates Tdp-43 M337V-associated mitochondrial morphology changes. Using confocal microscopy, mitochondrial structure was visualized in NSC-34 cells expressing Tdp-43 M337V. The expression of this particular mutant leads to smaller mitochondria compared with that of non-treated cells transfected with empty control vectors, in accordance with previous reports (Fig. 7A and B) (46,93). Similarly, mitochondria were significantly smaller in cells expressing Tdp-43 M337V after oxidative stress treatment (Supplementary Material, Fig. S4A). In addition, a significant reduction in the total number of mitochondria was observed in cells over-expressing Tdp-43 M337V both in non-treated and treated conditions (Fig. 7B and Supplementary Material, Fig. S4B). We then tested if over-expression of Oxr1-C combined with Tdp-43 M337V restores normal mitochondrial morphology. In untreated cells, over-expression of Oxr1-C significantly increases mitochondria size to the level of cells transfected with empty vectors, and although mitochondria number is increased in cells co-transfected with Oxr1-C, it does not return to control levels (Fig. 7B). In arsenite-treated cells, Oxr1-C co-transfected with Tdp-43 M337V mutant increases both mitochondrial size and number, but without reaching significance (Supplementary Material, Fig. S4A and B).

We next tested whether the changes in mitochondrial morphology associated with the Tdp-43 M337V mutant affected mitochondrial function by analysing mitochondrial respiration. NSC-34 cells were transfected with various ALS-Tdp-43 mutants, and mitochondrial oxygen consumption was measured. Of all the Tdp-43 mutants tested in non-treated and oxidative stress conditions, only cells over-expressing Tdp-43 M337V show a significant decrease in mitochondrial respiration under non-treated conditions (Fig. 7C and Supplementary Material, Fig. S4C). Importantly, we found that over-expression of Oxr1-C with Tdp-43 M337V restores mitochondrial respiration to levels of empty vectors-transfected cells (Fig. 7C). Parallel studies were carried out with ALS-Fus mutants, and no changes in oxygen consumption were observed in cells transfected with either wild-type or ALS-mutant Fus in non-treated or arsenite-treated cells (Fig. 7C and Supplementary Material, Fig. S4D). Taken together, these data suggest that Oxr1-C improves certain aspects of mitochondrial dysfunction in ALS-associated Tdp-43 M337V mutant cells.

## Discussion

The purpose of this study was to understand the neuroprotective mechanisms of Oxr1 by utilizing a protein binding approach. We identified the ALS-associated proteins Tdp-43 and Fus as interactors, in addition to proteins involved in several key neuronal pathways. Importantly, we showed that Oxr1 could positively modify the deleterious cellular features of pathogenic TDP-43 and FUS mutations.

Using our unbiased proteomic approach, we have demonstrated for the first time that Oxr1 binds to proteins involved in the oxidative stress response. This is significant as it has been shown that Oxr1 plays an important role in these pathways, although the molecular mechanisms are unclear; particularly as the three-dimensional structure of the highly conserved TLDc domain did not reveal similarity to known antioxidant proteins (53). Thus, we investigated binding partners of both the longest (Oxr1-FL) and the shortest (Oxr1-C) TLDc-containing isoforms of Oxr1 for a comprehensive study. First, we showed that these two isoforms are not necessarily co-regulated under oxidative stress conditions, suggesting that they have independent functions. This was confirmed by our proteomic analysis: Oxr1-FL and Oxr1-C share some common binding partners, but many interactors are isoform-specific. It is also noteworthy that the majority of Oxr1-C binding partners were identified under oxidative stress conditions, while Oxr1-FL appears to interact with the same proteins both in non-treated and in sustained exposure to oxidative stress; this suggests that Oxr1-FL is functionally independent of cellular oxidative stress levels, while Oxr1-C is primarily functional under oxidative stress. Thus, we have shed new light on the function of the Oxr1-C isoform; this is important as we demonstrated previously that Oxr1-C is sufficient to prevent oxidative stress-induced neuronal cell death both *in vitro* and *in vivo* (51).

Our *in silico* pathway analysis of Oxr1-FL and -C binding partners identified significantly associated pathways through which Oxr1 carries out its neuroprotective function, namely the mTOR signalling, and regulation of its downstream proteins (p70S6K and eIF4), as well as the EIF2 signalling; these pathways are well established as essential for the regulation of the stress response and cell survival (56–58). Furthermore, Oxr1-C specifically binds to proteins involved in the Nrf2-mediated oxidative stress response, another key antioxidant defence pathway (94). Our study also identified a novel potential function for Oxr1 in the inflammatory response, which is in line with findings from a recent study in *Drosophila,* which demonstrated that the TLDc domain-containing protein Mustard (*Mtd*) regulates the innate immune response and tolerance to infection (95). Therefore, we have confirmed the role of Oxr1 in the oxidative stress response and provide the first mechanistic insights into Oxr1 function.

We showed previously that OXR1 is induced in biopsy samples from ALS patients as well as in the spinal cord of pre-symptomatic SOD1 G93A ALS mice (51), so we hypothesized that OXR1 plays a role in the pathophysiology of ALS. Therefore, it was of particular interest that from our proteomic screen, we identified six binding partners of Oxr1 that present either a variant or a mutation associated with ALS: FUS, TDP-43 as well as hnRNPA2B1, hnRNPA1, EWSR1 and PRPH (1,11,63,96–99). Here, we focused on the process regulating Fus and Tdp-43 localization and aggregation and showed that Oxr1-C restores nuclear localization of Fus and Tdp-43 mutants and reduces cytoplasmic aggregation through a pathway independent of proteasome- and autophagy-mediated degradation, but dependent on Prmt1-modulated arginine methylation. Interestingly, although cytoplasmic aggregation of over-expressed Fus does not significantly differ even when proteasome and autophagy are inhibited, we demonstrated for the first time that ALS-Fus mutants are degraded in a pathway dependent on both proteasome and autophagy. This difference of degradation for wild-type and ALS mutant Fus suggests that ALS-associated mutations in Fus may alter the normal pathways by which Fus is degraded, thereby facilitating recruitment to cytosolic inclusions, particularly under increased levels of reactive oxygen species (ROS). Next, we identified and confirmed Prmt1 as a binding partner of Oxr1-C and Fus, as well as Tdp-43. Recent studies have found that arginine methylation of Fus by Prmt1 regulates Fus localization (28–33). Interestingly, as previously reported, we showed that inhibition of Prmt1 after the transfection of Fus constructs results in increased recruitment of wild-type and ALS-Fus mutants to cytoplasmic aggregates (28–29). There are conflicting reports about whether Prmt1 and arginine methylation increase or reduce cytoplasmic Fus and Fus-positive cytosolic inclusions (28–33), but we demonstrate here that the loss of Prmt1 function leads to increased aggregation of Fus in the cytoplasm, and that Oxr1-mediated reduction in wild-type and ALS mutant Fus aggregation requires Prmt1 function. Furthermore, although inhibition of Prmt1 activity does not affect recruitment of wild-type and ALS mutant Tdp-43 to cytoplasmic aggregates, over-expression of Oxr1 no longer reduces Tdp-43-positive cytosolic inclusions when Prmt1 function is inhibited. Taken together, these results implicate a Prmt1-dependent pathway downstream of Oxr1 function under oxidative stress, and suggest that this pathway also regulates Fus nucleo-cytoplasmic localization without altering arginine methylation of Fus.

The exact mechanisms by which Oxr1-C induces Fus and Tdp-43 relocalization to the nucleus are still unclear. Only mutants with a preserved binding for Oxr1-C, with at least an affinity 80% similar to wild-type proteins, relocate to the nucleus when cells are co-expressed with Oxr1-C, thus suggesting that binding of Fus, and Tdp-43 with Oxr1-C is necessary for this process to take place. Interestingly, our proteomic approach identified a number of proteins that, together with Oxr1-C, could convey this function. Transportin (or Ran) and Eef1a1, two nuclear transport proteins, were identified as Oxr1-C binding partners. Transportin is essential for the translocation of proteins through the nuclear pore complex (100) and Eef1a1, a known Fus and Tdp-43 binding partner, is part of the nuclear protein export machinery (101). Thus, Oxr1 could modulate the binding between Fus, Tdp-43 and nuclear transport proteins, which in turn would control the shuttling of Fus and Tdp-43 between the nucleus and the cytoplasm; further studies are required to test this model.

We specifically studied the effect of Oxr1-C over-expression on mitochondrial morphology defects and dysfunction induced by Fus and Tdp-43 mutants. Indeed, studies have found Oxr1 expression in mitochondria, which suggests a role for Oxr1 in regulating mitochondrial morphology, function and mitochondrial DNA replication (52,60). Our *in silico* analysis of Oxr1 binding partners identified RNA-post-transcriptional modification as a downstream function regulated by Oxr1-C, and given the known function of Fus and Tdp-43 as splicing regulators, we assessed the splicing of genes that modulate mitochondrial morphology and function. Interestingly, we found that co-expression of Oxr1-C with Tdp-43 M337V rescued the splicing changes of a mitochondrial fission gene, *Mtfr1,* associated with Tdp-43 M337V specifically under oxidative stress, which suggests a function for Oxr1 in mitochondrial fission and replication. A recent study demonstrated that co-expression of the mitochondrial fusion protein mitofusin 2 (Mfn2) with Tdp-43 M337V mutant reduced the Tdp-43 M337V-induced mitochondrial morphological and functional abnormalities (46). However, unlike our current study, it did not rescue Tdp-43 M337V cytoplasmic mis-localization or the associated splicing defects (46). Moreover, from our proteomic approach, we identified mitochondrial Atp5b and Slc25a5 as Oxr1-C binding partners. Atp5b is a subunit of the mitochondrial ATP synthase, and Slc25a5 is a mitochondrial adenine nucleotide translocator (102,103). Considering the coupling between the electron transport chain, oxidative phosphorylation and ATP production, and given that over-expression of Oxr1-C modulates oxygen consumption of mitochondria, further studies will be required to investigate whether this interaction directly tunes ATP synthase activity. This would in turn control both the mitochondrial oxygen consumption and the production of ROS, whose accumulation leads to oxidative stress.

In conclusion, we provided new insight into the function of Oxr1. We identified binding partners of two of Oxr1 isoforms as well as new functions for this protein. Our study identifies Oxr1 as a modifier of three major cellular pathological features of ALS associated with Fus and Tdp-43 mutations: mis-localization and aggregation of mutant proteins, splicing defects and mitochondrial abnormalities. Taken together, these findings suggest that Oxr1 serves as a potential therapeutic target for ALS and other neurodegenerative disorders characterized by TDP-43 or FUS pathology.

## Materials and Methods

### Constructs

Constructs pCX-Oxr1-FL-HA and pCX-Oxr1-C-HA were generated using a pCAGGS-derived vector as previously described (51). Oxr1-FL corresponds to protein accession number NP_570955 and Oxr1-S is based on NP_001123636 with an additional 28 amino-acid second exon that is shared with Oxr1-FL. pCDNA3-Fus-Myc and pCDNA3-Tdp-43-FLAGx3 were derived from full-length coding sequences (CCDS21886.1 and CCDS38970.1) cloned with their respective C-terminal tags. Mutant clones were generated by site-directed mutagenesis (Quikchange, Agilent) or by the addition of the mutant nucleotide to the cloning primers. For Prmt1, the full-length coding sequence (CCDS57547.1) was cloned into pcDNA3.1 with either a C-terminal HA-tag or FLAG-tag.

### Cell culture, transfections and treatment

Negroid cervix epitheloid carcinoma (HeLa), Neuro2a (N2a) and mouse motor neuron-like cell line (NSC-34) were grown in DMEM supplemented with 10% FBS, 2 mm l-glutamine, 1 mm non-essential amino acids and 1% ampicillin–streptomycin. For HeLa and N2a cells, cells were transiently transfected using FuGENE 6 (Roche Diagnostics). NSC-34 cells were transfected using Magnetofection (Oz Biosciences) as per manufacturer's instructions. One day after transfection, cells were treated with H_2_O_2_ (Sigma) or sodium arsenite (Sigma) for dose and duration indicated in text. For proteasome inhibition experiments, cells were treated with 10 µm MG-132 (Calbiochem) for 4 h before subsequent oxidative stress treatments. For inhibition of autophagy-lysosomal degradation or arginine methylation, cells were treated with 1 µm Concanamycin-A (Santa Cruz) or 100 µm AMI-1 (Sigma), respectively, for 12 h before subsequent oxidative stress treatments.

### Protein analysis

To study interactors of Oxr1 (both full length and short isoforms), N2a cells were transfected with the HA-tagged Oxr1 full length or short isoform in pCX vector. Cells were transfected for 24 h. After H_2_O_2_ treatment, cells were washed twice with cold PBS and lysed with lysis buffer [50 mm Tris (pH = 7.5), 150 mm NaCl, 1% Chaps] complemented with a cocktail of protease inhibitors (Roche). Lysates were cleared by centrifugation (30 min, 4°C, 16 000*g*) and 50 μl of pre-equilibrated EZview Red Anti-HA (Sigma) was added to the lysates and incubated overnight at 4°C. Immunoprecipitated proteins were then washed five times with lysis buffer. Proteins were resuspended in NuPAGE LDS sample buffer (Life Technologies) and boiled for 5 min. Samples were run through the stacking gel of a NuPAGE gel (Life Technologies) and the entire band was cut and sent for trypsin digestion and analysis by LC-MSMS sequencing identification (Dunn School of Pathology, University of Oxford). The list of protein targets were analysed using Ingenuity software. The *P*-values calculated by the software are a measure of the likelihood that the association between the set of proteins identified in the experiment and a given process or pathway is due to random chance. The *P*-value for a given function is calculated by considering the number of functional analysis molecules that participate in that function and the total number of molecules that are known to be associated with that function in the Ingenuity Knowledge Base.

For immunoprecipitation of HA, MYC and FLAG-tagged proteins to confirm binding targets, cleared protein extracts were incubated overnight at 4°C with EZview Red Anti-HA, MYC or FLAG Affinity Gel beads (Sigma), with no pre-clearing step. Immunoprecipitated proteins were washed three times with lysis buffer and resuspended in 20 μl of sample Laemmli buffer (Biorad) and protein extracts were run on a 4–12% or 10% pre-cast gels (GE Healthcare). Western blots were probed with the primary antibodies described below and with secondary antibody anti-HRP rabbit or mouse (Life Technologies) using ELC (Amersham) and Clean Blot IP detection reagents (ThermoScientific) with an ImageQuant LAS4000 (Amersham) or a Compact X4 (Xograph Imaging system).

Primary antibodies used were as follows: mouse anti-FLAG (Sigma, 1:4500); rabbit anti-FUS (Bethyl, 1:10 000); rabbit anti-HA (Sigma, 1:10 000); mouse anti-mono and dimethyl arginine (Abcam, 1:1000); mouse anti-Myc (Sigma, 1:2500); rabbit anti-Oxr1 [(51), 1:1000]; rabbit anti-PRMT1 (Millipore, 1:1000) and mouse anti-β-actin (Sigma, 1:6000).

### Immunofluorescence

HeLa, N2a and NSC-34 cells were rinsed with PBS, fixed with 4% paraformaldehyde for 15 min, and incubated with 0.1% Triton 100X for 20 min at room temperature. Primary antibodies against TIA-1 (Santa Cruz Biotechnology, 1:100), COX4 (Abcam, 1:500), DCP1A (Sigma, 1:200), FLAG (Sigma, 1:100); FUS (Bethyl, 1:250); rabbit HA (Sigma, 1:100); Myc (Sigma, 1:100) and TDP-43 (Proteintech, 1:350) were incubated from 1 h to overnight at room temperature or 4°C depending on antibody. After three washes with PBS, coverslips were incubated with Alexa anti-mouse, rabbit, rat, or goat secondary antibodies (Invitrogen, 1:700) for 1 h or overnight. Coverslips were subsequently washed and mounted in DAPI medium (VectorLabs). Images were captured by Axioplan-2 Imaging fluorescent microscope (Carl Zeiss) or TCS SP5II confocal microscope (Leica Microsystems CMS GmbH).

### Quantitative RT–PCR

RNA was extracted using TRIzol reagent (Invitrogen) followed by purification and DNAse I-treatment on a mini column (Qiagen) as per the manufacturer's instructions. RNA was reverse transcribed using reverse transcriptase (Roche) and cDNA was used in a quantitative RT–PCR reaction using SYBR Green PCR master mix (Applied Biosystems) and a Step One real-time PCR machine (Applied Biosystems). *Gapdh* was used as control gene (Table 2).

**Table 2.** Primers used in this study

qRT–PCR	
Oxr1 FL-F	CAGTCGTGACTGGACAGGT
Oxr1 FL-R	ATGGGCTACATCTGGAGTCG
Oxr1 C-F	CCATAAATACACTCTGGTAGTGTCG
Oxr1 C-R	TTTGGTCGGAAAGATTCAGG
Tdp-43-F	CTCCCCTGGAAAACAACTGA
Tdp-43-R	AAAGCCAAACCCTTTCGAGT
Fus-F	CCTAGCAGCACCTCAGGAAG
Fus-R	CGTAGTTTGTTGCTGTCCA
Splicing	
Mapt1-F	TCCCCCTAAGTCACCATCAG
Mapt1-R	GCCAATCTTCGACTGGACTC
Opa1-F	GGAGAAACAGCATTTCGAGC
Opa1-R	GCAGAAGTTCTTCTTGAAGTTGG
Mtfr1- F	TGATTCAGTGTCCAAGAGTTCAA
Mtfr1-R	CTTCTGCAGGGCCTCCTCGCT
Tab1b-F	CCCCAACACCAAGATCAACT
Taf1b-R	AGGCCTGTTTGCTCTTCTGA
Tia1-F	TGAAAGTGAATTGGGCAACA
Tia1-R	TGCCCTTTAGGTGGTGAAAG
Scl1a2-F	ACCGAATGCAGGAAGACATG
Scl1a2-R	GGCTGAGAATCGGGTCATTA
Eif4h-F	ACTTCGTGTGGACATTGCAG
Eif4h-R	CCCCCTACCCCCTAAGAAGT

### Molecular analysis of alternative splicing

RNA was extracted from transfected NSC-34 cells using a mini column (Qiagen) as per the manufacturer's instructions and subjected to DNAse I treatment (Qiagen). cDNA was generated using reverse transcriptase and oligo dT primers (ThermoScientific). To quantify splicing, cDNA was used in a PCR reaction with Red Taq enzyme (Sigma) and run on a 2% agarose gel and visualized using an Alphalmager 3400. The forward and reverse primers for each reaction were located in exons situated on either sides of an exon indicated in bracket on the figures (Table 2). The intensity of the bands was quantified using ImageJ software (NIH) and a ratio of inclusion (top band) to exclusion (bottom band) of indicated exon was calculated (in/ex).

### O_2_ consumption assay

Oxygen consumption assay was carried out as per the manufacturer's protocol (Luxcel Biosciences, Ireland) with minor changes. NSC-34 cells were plated at ∼3.3 × 10^5^ cells/cm^2^ in culture media in 96-well Falcon plates. Twenty-four hours after transfection, culture medium supplemented with MitoXpress probe at a final concentration of 62.5 nm was added to the wells. To each well, 100 μl of pre-warmed high sensitivity oil was quickly added immediately before the measurement and the plate placed in a pre-warmed (37°C) plate reader (FLUOstar Omega microplate reader, BMG Labtech). The oxygen probe signal was measured using a time-resolved mode at excitation and emission wavelengths of 380 and 650 nm, respectively. The slope was calculated as the slope of the linear part of the oxygen consumption curve and the percentage was calculated using values for cells transfected with empty vectors as 100%.

### Mitochondria quantification

To quantify COX IV-stained mitochondria in NSC-34 cells, confocal images (1024 × 1024 pixels) were captured using a Leica TCS SP5II confocal microscope (×63 oil objective, zoom: 7 or 9; scan speed: 200 Hz) with a LAS AF software. Images were then exported and processed with NIH ImageJ FIJI software. The entire range of grey values (0–255) was covered by contrast optimization. Then, binary images of mitochondria were generated. The number of mitochondria per cell and the average area of a mitochondrion were subsequently evaluated by particle analysing tool (size in pixcel^2^: 10-infinity; circularity: 0.00–1.00). Thirty to 40 cells per condition were analysed.

### Statistical analysis

Results were analysed using Prism software. The difference between wild-type and mutant or between the various treatments was compared using a one- or two-way ANOVA test followed by Dunnett's multiple comparison test or Bonferroni's multiple comparison test when appropriate. *P*-values of <0.05 were considered significant. Data were expressed as mean ± SEM.

## Supplementary Material

Supplementary Material is available at *HMG* online.

## Funding

This work was supported by the UK Medical Research Council (to K.E.D.); the European Research Council (PAROSIN to P.L.O.) and the Clarendon and US–UK Fulbright Scholarships (to K.X.L.). Funding to pay the Open Access publication charges for this article was provided by the University of Oxford RCUK Open Access Block Grant.

## Supplementary Material

Supplementary Data
