# Supplementary material for: Oxr1 improves pathogenic cellular features of ALS-associated FUS and TDP-43 mutations
Source: Hum Mol Genet. 2015 Mar 19;24(12):3529–44. doi: 10.1093/hmg/ddv104 (PMC4498158; doi:10.1093/hmg/ddv104)
Supplement: Supplementary Data [file supp_24_12_3529__index.html]

Oxr1 improves pathogenic cellular features of ALS-associated FUS and TDP-43 mutations — Oxr1 improves pathogenic cellular features of ALS-associated FUS and TDP-43 mutations — Oxr1 improves pathogenic cellular features of ALS-associated FUS and TDP-43 mutations — Supplementary Data 

# Oxr1 improves pathogenic cellular features of ALS-associated FUS and TDP-43 mutations

## Supplementary Data

Supplementary Data

**Files in this Data Supplement:**

- Supplementary Data - Doc file
