# Supplementary material for: Oxr1 improves pathogenic cellular features of ALS-associated FUS and TDP-43 mutations
Source: Hum Mol Genet. 2015 Mar 19;24(12):3529–44. doi: 10.1093/hmg/ddv104 (PMC4498158; doi:10.1093/hmg/ddv104)

**Supplementary Figure legends**

**Figure S1. Wild-type and ALS-mutant Fus and -Tdp-43 are localised to stress granules under oxidative stress conditions in HeLa cells.** (A) Representative images of co-localisation of cytoplasmic aggregation of Fus, and Tdp-43 wild-type (wt) and mutants with TIA-1-positive stress granules under arsenite treatment. Scale bars: 50 μm. (B) Quantification of cells forming aggregates under arsenite treatment when co‐transfected with wt and mutant Fus and Tdp-43 with either empty vector (pCX) or Oxr1‐C. Statistical significance was determined by two‐tailed unpaired Student’s t‐test (n=3). **p<0.01, and ***p<0.001.

**Figure S2. Binding of Oxr1-C with Fus and Tdp-43 does not require Prmt1 activity.** (A) Co-IP of Oxr1-FL and Prmt1 in co-transfected N2a cells shows that Oxr1-FL does not bind Prmt1. (B) Methylation of endogenous Fus is decreased, but methylation of transfected Fus is not altered after treatment with AMI-1, a potent inhibitor of Prmt1 function. (C-D) Inhibition of Prmt1 activity by AMI-1 does not alter binding between Oxr1-C and Fus (C) or Tdp-43 (D).

**Figure S3. Over-expression of Fus and Tdp-43 wild-type and ALS-associated mutants in motor neuron-like cells does not affect splicing of a subset of mitochondrial and oxidative stress-response genes.** (A-B) NSC-34 cells were transfected with either Fus or Tdp-43 wild-type (wt) and mutants with either pCX empty vector or Oxr1-C for 24 hours. (A) qRT-PCR demonstrating that levels of Fus, Tdp-43 and Oxr1-C were not different in over-expressing cells. (B) Cells were treated with arsenite for 4 hours. The ratio of the inclusion to exclusion ratio (in/ex) of the exon(s) indicated in brackets was calculated and used as a measure of gene splicing. Statistical significance between non-treated and treated conditions was determined by two-way ANOVA followed by Bonferroni's test (n=3-6).

**Figure S4. Motor neuron-like cells over-expressing Tdp-43 M337V mutant present shorter mitochondria, but their oxygen consumption is not affected under oxidative stress.** (A-B) The average mitochondrial area (A) and average number of mitochondria (B) were quantified. Mitochondria in cells transfected with Tdp-43 M337V and treated with arsenite for 30 minutes were shorter and fragmented as compared to cells transfected with empty vectors. (C) Oxygen consumption was quantified in NSC-34 cells transfected with the indicated vectors for 24 hours and treated with 0.5mM arsenite for the entire duration of the assay. The slope was calculated as the slope of the linear part of the oxygen consumption curve and compared to level in cells transfected with the empty vectors. Statistical significance was determined by one-way ANOVA (n=3-6).


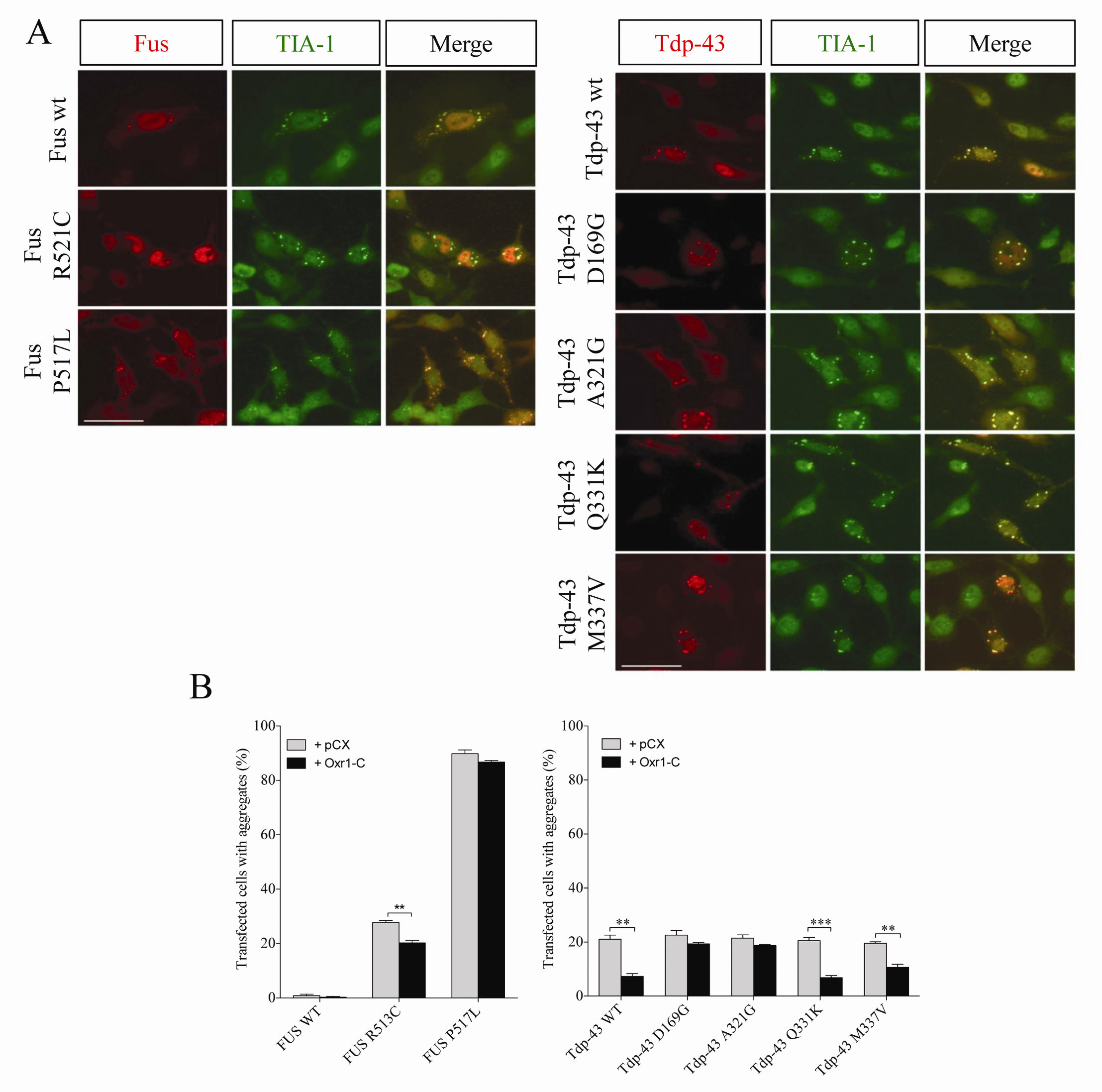


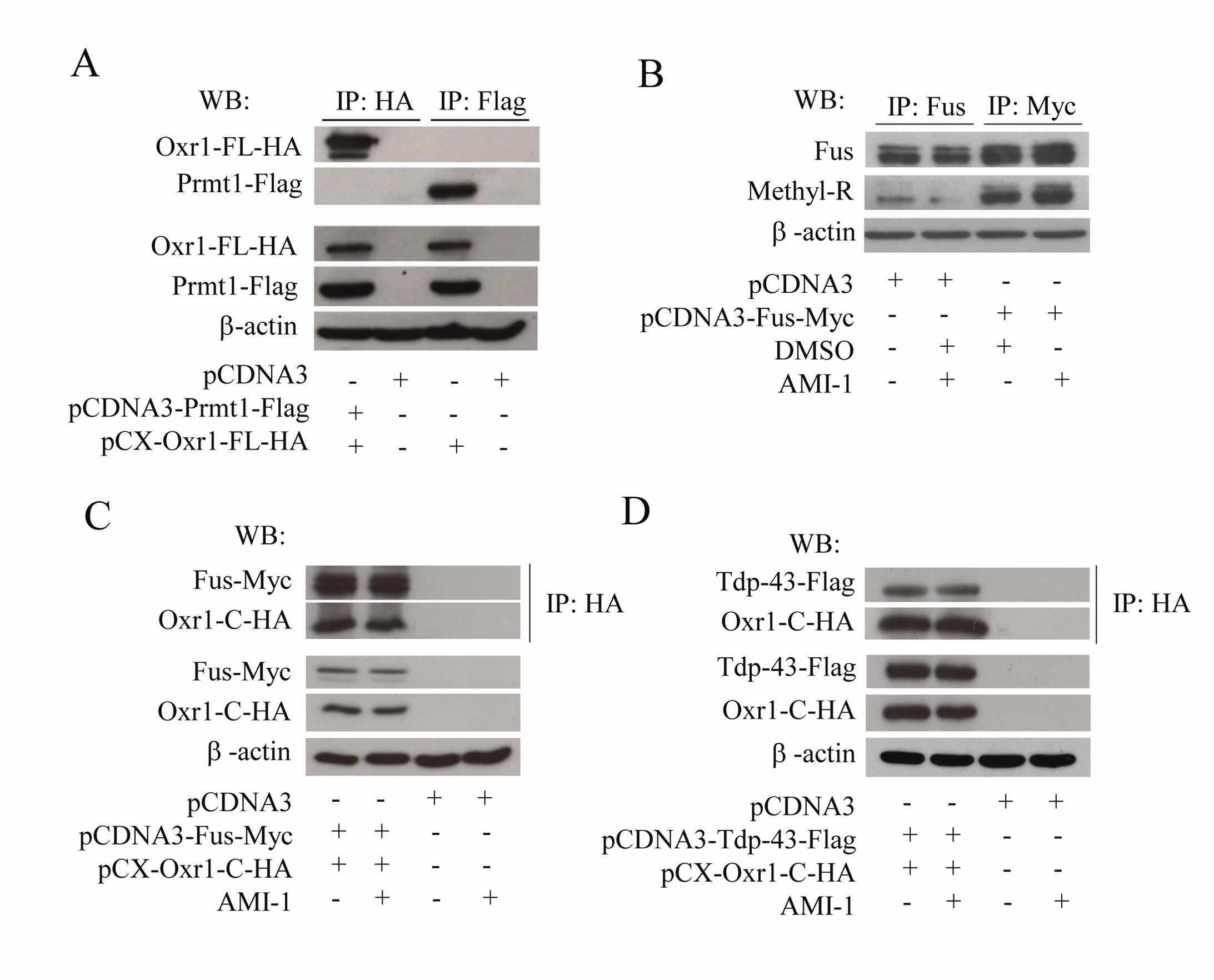


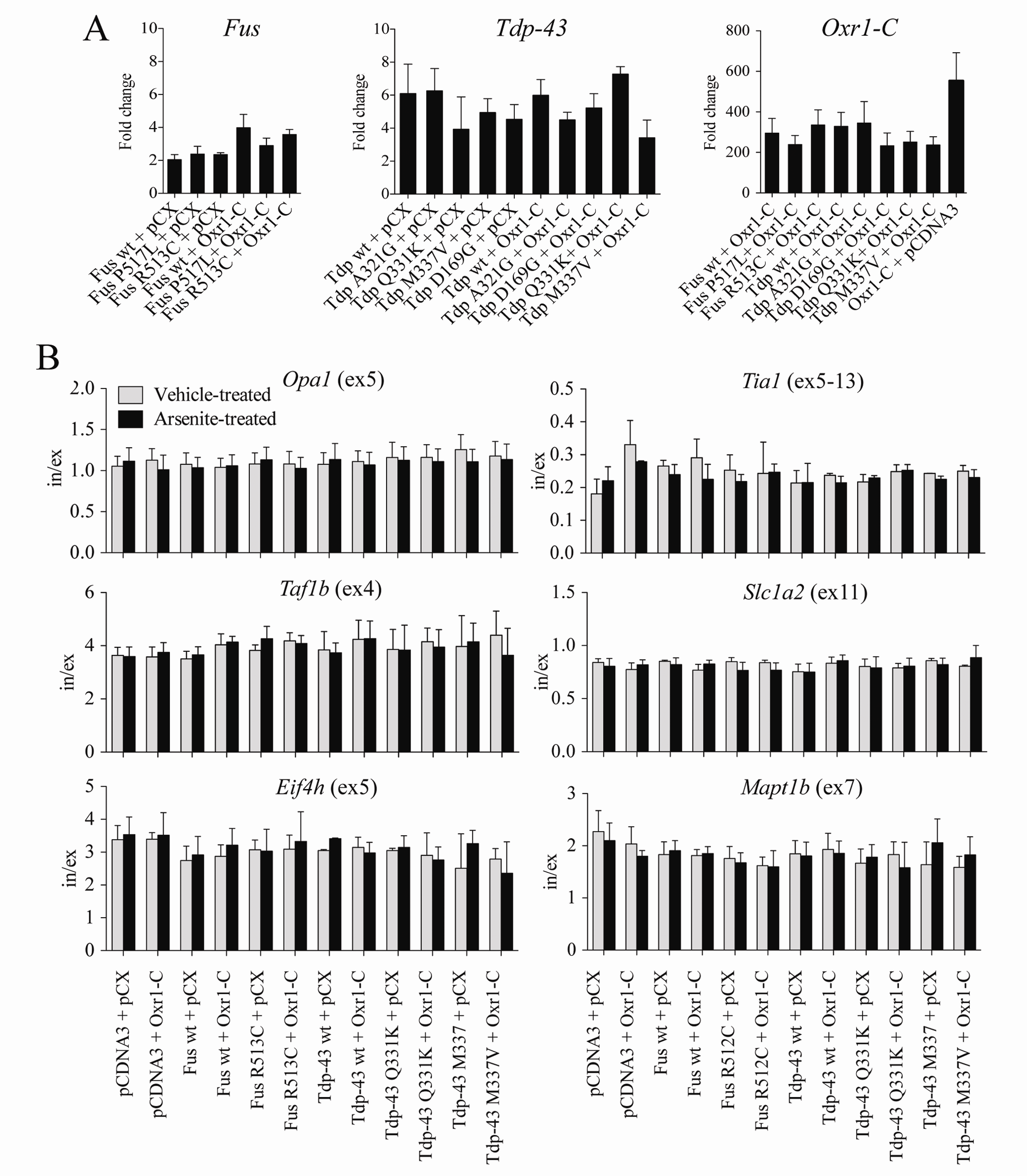


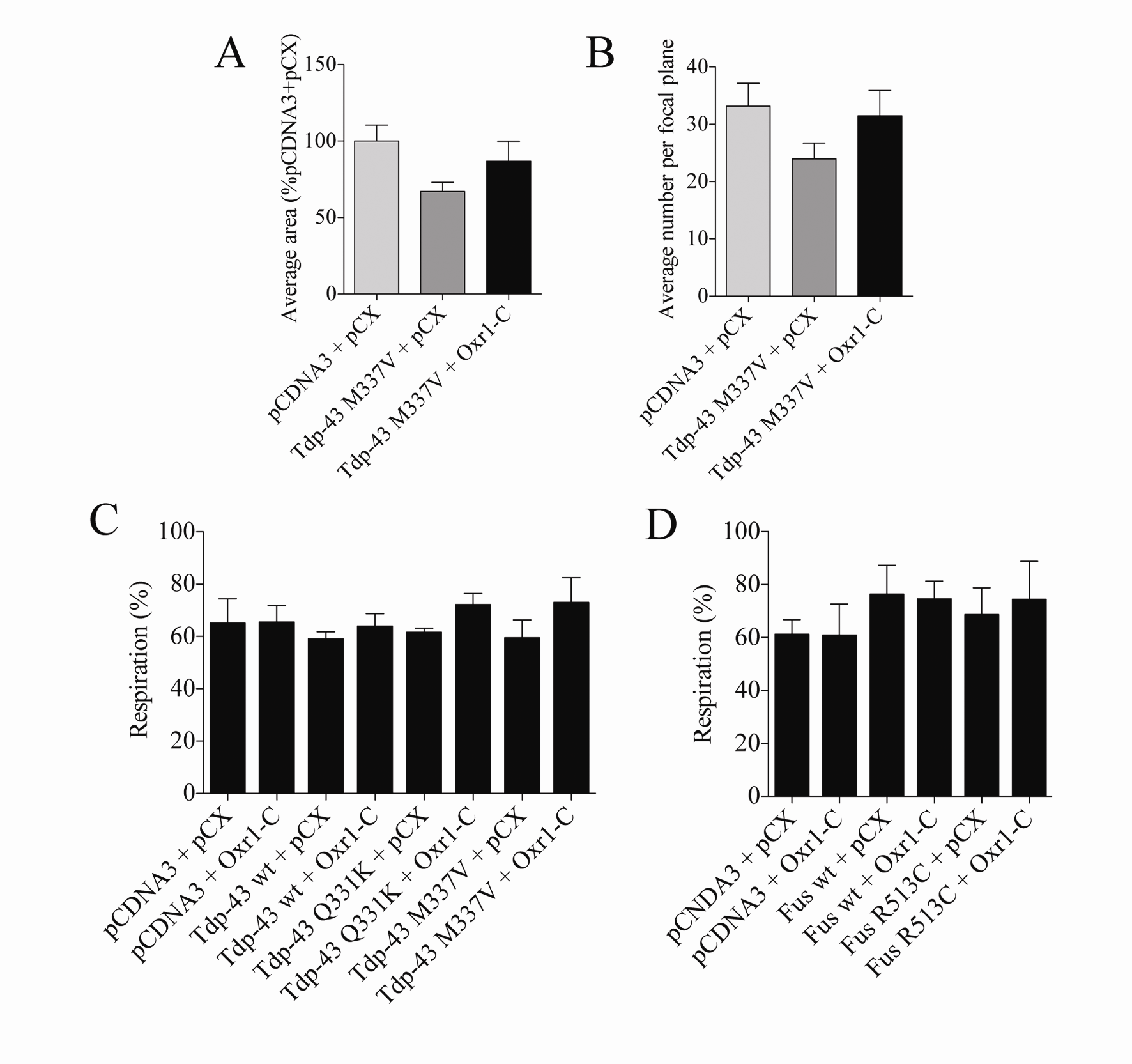

Supplement: Supplementary Data [file supp_ddv104_ddv104supp.doc]
